# Before the COVID-Vaccine—Vulnerable Elderly in Homecare

**DOI:** 10.3390/nursrep12020027

**Published:** 2022-04-07

**Authors:** Liv Wergeland Sørbye, Else Vengnes Grue, Sophie Hogeveen

**Affiliations:** 1 Department of Health, VID Specialized University, NO-0319 Oslo, Norway; ven-gr@online.no; 2 Department of Health Research Methods, Evidence & Impact, McMaster University, Hamilton, ON L8S 3L8, Canada; hogevees@mcmaster.ca

**Keywords:** homebound elderly, COVID-19 screening, homecare, loneliness, insecurities about the future

## Abstract

Background: At the beginning of 2020, the COVID-19 virus was spreading all over the world. Frail elderly were at risk for illness and death. Isolation seemed to be the best solution. The aim of this paper was to describe how the lockdown affected elderly homecare patients. Methods: We used an international self-reported screening instrument built on well-documented risk factors adapted to COVID-19. We considered ethical, legal, and practical concerns. The research included telephone interviews with 30 homecare patients. Results: Seventy percent lived alone. Seventy-three percent of the sample suffered from major comorbidity. Cardiovascular disorder was the most frequent diagnosis. Nineteen (63.3%) needed help for personal care. Several of the participants were lonely and depressed. The homecare teams struggled to give proper care. The health authorities encouraged the population to reduce their outside physical activities to a minimum. The restrictions due to COVID-19 affected daily life and several respondents expressed uncertainties about the future. Conclusions: It is important to describe the patients’ experiences in a homecare setting at the initiation of lockdowns due to COVID-19. The isolation protected them from the virus, but they struggled with loneliness and the lack of physical contact with their loved ones. In the future, we need to understand and address the unmet needs of elderly homecare patients in lockdown.

## 1. Introduction

In the early spring of 2020, the coronavirus disease (COVID-19) disaster was spreading from one continent to another. The World Health Organization realized that they had little insight to offer the jurisdictions now grappling with this novel disease and the thousands of people who were infected. However, on their website, they advised the public and updated information about the coronavirus disease (COVID-19) [1] Those infected often had trouble explaining how and where they had contracted the virus. There was a huge lack of the proper knowledge, expertise, equipment, and medical treatment to prevent this new viral disease. The situation in Norway was no different; we were not prepared. The health authority’s advice led the government to a national lockdown beginning on 12 March 2020.

Older people are particularly at risk of developing severe illness if infected due to physiological changes that come with aging and potential underlying health conditions [2,3,4,5]. Furthermore, it may be difficult to differentiate symptoms of COVID-19 from other age-related ailments such as lung problems and general frailty. The residents in nursing homes are very vulnerable, and the spread of the COVID-19 virus in these settings was overwhelming [6,7,8,9,10]. The same was true for homecare agencies, which provide essential medical and supportive services to community-dwelling older adults [11]. Home-based care is an alternative to facility-based care, and often seems to be the best and most desirable choice [12,13]. In Norway, nursing homes and community homecare are important parts of service to the elderly and are publicly funded. The elderly pay a part of their pension, about 85%, for full time residence and care in a nursing home. Community homecare is free of charge. However, there is a deductible to pay for home service for those with an income above a certain minimum (https://lovdata.no/dokument/SF/forskrift/2011-12-16-1349, accessed on 5 May 2021).

In homecare, as in society in general, physical distancing and limiting in-person interactions became important to prevent infection during the pandemic. Because of the invisible virus, the homebound elderly were isolated and had reduced opportunities to leave their apartment, becoming isolated. Furthermore, due to the fear of spreading COVID-19, nursing visits were reduced [14,15], which led to unmet needs and challenged both the relatives of the elderly and health authorities [16]. There were also staffing and training-related challenges as the public health measures had an impact on the training of new nurses in the homecare setting. Practical studies in home nursing are mandatory and include learning objectives such as strict hand hygiene, facemask wearing, and distancing, which require application in the home setting. Nursing students were also forced to continue their studies online.

As a result of all the public health measures in place, older adults were at greater risk of decline in function, cognition, or mood because these unmet needs were not known or addressed until they reached a crisis point [17].

The aim of this study was to identify possible symptoms and early signs of decline with a COVID screening instrument, and describe how the lockdown affected elderly homecare patients.

## 2. Methods

The study design was a cross-sectional mixed methods approach. We invited elderly homecare patients to participate in an interview where data were collected through a researcher-administered structured self-report survey with open-ended responses elicited linked to each question.

### 2.1. Procedures and Participants

The local homecare office distributed information letters in late spring 2020, prepared by the study authors (LWS, EVG) to older adults enrolled in a homecare team in Oslo requesting their participation in a telephone call to respond to questions about their health, well-being, nursing care, and the impact of the lockdown on their lives. Their homecare nurses obtained informed consent and shared the telephone numbers of participants with the first author and obtained an appointment for a telephone interview.

Unfortunately, as COVID-19 accelerated after a few months, the nurses were not able to use their time to explain the study and screening information. After the summer vacation, we tried to activate nursing students for the task. However, the elderly people were too scared to meet more nurses than what was strictly needed. By then, we had interviewed 30 persons, 33% of the total number of users of the homecare office. As far as the staff could tell, these persons were representative of their clients as a whole in terms of age, gender, and need of care.

### 2.2. interRAI COVID-19 Vulnerability Screener

Shortly after the outbreak of COVID-19, the interRAI organization (www.interrai.org, accessed on 15 December 2021) developed a web-based screening tool to identify vulnerable elderly and adults with disabilities living in the community who were at risk during the pandemic and to determine individuals who required further assessment and/or intervention. InterRAI is a non-profit network of international researchers, clinicians, and policy experts that develops and validates comprehensive assessments to identify the strengths, preferences, and needs of vulnerable persons with complex health conditions [18,19,20]. The interviews started around the beginning of June. The interviewer (LWS) verbally administered the structured interRAI COVID-19 Vulnerability Screener (CVS) survey over the phone, and elicited open-ended responses linked to each CVS question. The length of the telephone interview was 15–30 min.

The interRAI COVID-19 Vulnerability Screener (CVS) consists of key questions in relation to health status, cognition, functioning, mood, and need for help with finances, access to food and medications, and use of health services. The CVS is a self-reporting survey, answered by respondents according to their perspective, and, if required, with support from a family member or caregiver. The survey may be completed in-person or over the phone and administered by a layperson. The survey contains 27 items, some with sub-items, in a closed-ended multiple-choice format. The CVS was developed by including self-reporting questions that were previously validated through their inclusion in two pre-existing interRAI instruments: (a) the interRAI Contact Assessment (CA) [21] and (b) the interRAI Check-Up (Self-Reported version) (CU) [19,22]. Both pre-existing tools have been tested and used with vulnerable community-dwelling older adult populations in jurisdictions in Canada and South Africa and found to have a sufficient level of validity and reliability. While the current combination of questions included in the CVS is unique, all the questions included were lifted from the CA and CU “as is”. The questions were chosen based on clinical relevance in consultation with community care providers and physicians.

The software that hosted the web-based CVS (developed by Raisoft Ltd, Kokkola, Central Ostrobothnia, Finland, and provided free for the duration of the pandemic) automatically generated a risk profile report.

#### 2.2.1. COVID-19 Symptoms

The screener intended to identify the presence of twelve COVID-19 symptoms: dry cough, sore throat, fever, chest pressure or pain, feeling confused, difficulty waking up, diarrhea, vomiting or abdominal pain, chills, headache, loss of taste/smell, or shortness of breath, and fatigue. These symptoms were chosen based on a review of self-assessment tools for COVID-19 released by the World Health Organization (WHO), Health Canada, and the Center for Disease Control (CDC). The COVID-19 Symptoms Flag indicates that the respondent reported one or more of the possible symptoms of COVID-19. The number of COVID-19 symptoms reported was captured in a count measure (COVID-SYM-CT; ranges from zero to twelve).

#### 2.2.2. Major Comorbidities Count (MCC)

The Major Comorbidities Count (MCC) indicates increased risk if infected with COVID-19 due to the presence of comorbidities (cardiac, lung, kidney, cancer, and neurological conditions) that have been identified by the CDC and WHO as associated with increased risk of death related to COVID-19 [23,24]. The algorithm was developed using over three million interRAI assessment records from nursing home and homecare patients in North America for validation. MCC score is calculated using the participants’ responses to questions about disease diagnoses and treatments/therapies. These questions have been validated as described above. Scores range from zero (low) to two (high risk).

#### 2.2.3. Activities of Daily Living Count (ADL-CT)

The Activities of Daily Living count (ADL-CT) is a summary score that captures the number of ADLs (including bathing, dressing lower body, locomotion, and personal hygiene) in which individuals require assistance (ranges from zero to four). If participants indicate that they require any help at all in each ADL, they will receive a score of one per ADL, up to a potential total score of four. The ADL questions have also been validated as described above.

#### 2.2.4. Assessment Urgency Algorithm (AUA)

The CVS assesses for frailty using the Assessment Urgency Algorithm (AUA), calculated using items for cognition, functioning, caregiver distress, self-rated health, mood, shortness of breath, and unstable conditions [18]. The AUA identifies individuals in urgent need of assessment, and ranges from one to six (higher scores indicate more urgent need). The AUA algorithm was first developed for use and validated in various care settings including primary care, homecare, and emergency departments in jurisdictions around the world [21]. The questions that inform the calculation of the AUA score in the CVS were validated as described above.

### 2.3. Analysis

The screener software, translated to Norwegian by the Finnish company Raisoft Ltd. (www.raisoft.fi, accessed on 15 April 2020), has embedded analytic functionalities to analyze the quantitative data. We used descriptive statistics to report symptoms, risk factors, and the need for care and medical help as measured using the CVS questions themselves.

We (LWS, EVG) conducted a descriptive analysis of the qualitative data obtained as noted from the open-ended responses participants made during the screening conversation. We read notes from the interviews for each participant, which were coded and discussed. We identified topics based on the data material and then discussed the topics’ relevance and organized the text according to central themes. The authors classified the notes from the interviews in groups.

### 2.4. Ethical Considerations

The project followed ethical guidelines for social sciences, humanities, law, and theology (https://www.forskningsetikk.no/en/guidelines/social-sciences-humanities-law-and-theology/guidelines-for-research-ethics-in-the-social-sciences-humanities-law-and-theology/, accessed on 10 April 2020). The Norwegian Center for Research Data reviewed and approved the proposal (ms-678259).

## 3. Results

The study included 30 persons, of whom 19 (63.3%) were female (see Table 1 for a description of the study sample). The mean age of the women was 83.0 years and of the men, it was 81.1 years.

Twenty-four (80%) of the participants answered all the interview questions by themselves. Six of the participants needed help from their spouse or another close relative. In these cases, they used the speaker on the phone and participated in the conversation. We organized the data collected using the CVS (Table 1) and additional data from the participants’ comments. Twenty-one (70%) of the participants lived alone. Eight (26.7%) lived with a spouse, and one lived with a daughter. Twenty-three (76.7%) of the sample lived with one or several other chronic diseases. Cardiovascular disorders and high blood pressure were the most frequent diagnoses. Seven patients (23.3%) had lung disease. The same number (*n* = 7, 23.3%) reported two or more possible COVID-19 symptoms. Twenty-two (73.3%) were at elevated or high risk of mortality due to the presence of comorbidities if they were to be infected with COVID-19.

About half of the participants reported feeling lonely occasionally or more often (*n* = 15, 50%) as well as feeling sad, depressed, or hopeless often or daily (*n* = 17, 56.7%). Twenty (66.7%) needed help with personal care such as bathing, showering, dressing, and toileting and were in urgent need of assessment as a result (AUA ≥5).

After we studied the results from the CVS, we carefully analyzed our notes from the open-ended responses from the participants. The notes described the participants’ experience while in lockdown. During the telephone interview, the participants talked about the positive and negative aspects of staying in their own home. We identified five main themes: (a) satisfied; (b) lonely; (c) depression; (d) worried about COVID-19 infection; and (e) death and dying (Table 2).

### 3.1. Satisfied

The participants mentioned different values that were important in their daily life. Three values mentioned the most often were close family/friends, communication via telephone or Internet, and the help from the homecare team.

Female, 82 years, AUA = 5 and COVID-SYM-CT = 0. “*I try to look at the bright side of life. I have two daughters and two grandchildren as well as good friends. We often talk on the phone. I call one friend every night before I go to bed*”.

Female, 90 years, AUA = 0 and COVID-SYM-CT = 0. She had medical treatment for coronary heart disease and high blood pressure. She spoke about her recent fall. “*I called 113* (the Norwegian alarm central for health emergencies). “*I did not want to bother my family in the middle of the night. However, I was cold lying on the floor with the window open. It took more than an hour before they arrived. Then they drove me to the emergency room. The diagnosis was a couple of rib fractures, and they took me home. I do not feel lonely. My family supports me well and the home care people are nice*”.

Female, 73 years, lived with her husband, AUA = 5 and COVID-SYM-CT = 2 with complicated care-needs. The patient said that she was satisfied with the homecare service. “*When the lockdown occurred, they organized a team of eight nurses for me. They worked in shifts day and night. They were skilled and had familiarized*”.

### 3.2. Loneliness

Fourteen [46.7%] people in the sample felt lonely. For some, the only visitors they received were nurses from the homecare team.

Female, 96 years, AUA = 6, and COVID-SYM-CT = 3. “*I feel lonely every day, even though I am living with my daughter. I am afraid of being abandoned*”. The daughter participated in the phone interview. Her mother was living in a nursing home until six months prior to the date of the interview. The daughter felt that she did not receive good and proper care and took her home. Since she was unable to move around, she stayed alone in a room all day.

### 3.3. Depression

Nineteen (63%) of the participants answered that they felt depressed due to the pandemic.

Female, 76 years, AUA = 6 and COVID-SYM-CT = 2. She also had multiple chronic disease diagnoses. She felt depressed and used antidepressants. “*My daughter “mini telephone calls” occasionally. She collects money from me once a month, for buying me food. Then she calls when she is outside my door. She does not dare to come in*”.

Female, 83 years, AUA = 6 and COVID-SYM-CT = 0. She had been in the hospital after a recent fall. She felt depressed and severe pain in her arms and in a knee. Her weight had decreased from 61 kg to 44 kg. She had home services 1 h and 13 min every day. She had physiotherapy but wanted more. She was unable to leave her home. “*It is problematic to use my arms to put on a jacket and proper shoes*”.

Male, 67 years, AUA = 6, COVID-SYM-CT = 0. “*I realize that I cannot do much outside the apartment. The danger of coronavirus means that there are no visits, neither am I able to talk on the phone*”.

### 3.4. Fear of COVID

The participants expressed that they were afraid of COVID-19 and therefore were reluctant to receive visitors. It was hard for them not to see their close family and friends.

Female 95 years. AUA = 4 and COVID-SYM-CT = 2. “*This corona time has been difficult. I used to visit the GP once a month. He has called me once during the last six months. I also miss my “exercise”. I used to train with a physiotherapist twice a week at a gym. The grandchildren used to visit regularly, not anymore. My daughter visited me only twice last month. We were sitting in a park at a distance of two meters*”. The participant shared that her family was more frightened about infection than she was.

Several of the participants complained that the nurses did not wash their hands properly and did not use sufficient equipment to prevent infection. They felt insecure that not all helpers liked to wear facemasks (see Table 2).

### 3.5. Death and Dying

Isolation and long days were hard for the elderly to endure. The future seemed uncertain and for several of them, the thought of death was not so frightening

Female, 94 years. AUA = 6 and COVID-SYM-CT = 0. “*I am not anxious. I receive help quickly when needed. I have a security alarm. I think I am going to die soon. I am calm. I hope I do not have a lengthy death process, but a short one*”.

Female, 84 years. AUA = 3 and MCC = 1. “*Usually I take a bath alone, but first I always call a friend who lives in this block. If I do not call her within a given time, she must come and lock herself in. I want an assurance that I will be found*”. She practices daily to get up on her own after falling to the floor. When her blood pressure becomes too low, she becomes dizzy, unstable, and falls easily. If something acute happens, she does not want to stay in a hospital. She hopes she can get help at home. She is not afraid to die, but would like palliative care and someone who could sit by her bed.

## 4. Discussion

The worldwide spread of the COVID-19 virus came as a surprise and in many countries, the infrastructure was not able to stop the pandemic. In the last few decades, Norway has been growing to a wealthy welfare state. The Sovereign Pension Fund—Foreign made it possible to give economical support to keep the country locked down (www.nbin.no, accessed on 22 February 2022). However, the money alone was not enough to stop the pandemic and the homecare sector was not immune from its effects. At the end of 2020, the infection had reached more than 5600 (1.3%) of the health workers in Norway [25]. Among the public, elderly people were disproportionately affected. Elderly people living at home were used to receiving help from close relatives or neighbors before the lockdown. The elderly also participated in activity and facility services provided by the community (www.ssb.no/en/helse/helsetjenester, accessed on 22 February 2022). This was also true for our respondents. In Norway, homecare nursing has expanded over the last decades. Of the inhabitants 80 years or older, about 30% were given home nursing or home help. We observed the same pattern in other Nordic countries. The pandemic changed their daily living—suddenly everything was closed. Isolation was one of the means to stop the spread of the COVID-19 virus and homecare nurses were their only “legal” visitors. The community care sector was not prepared, and they lacked qualified staff and infection control equipment. The turnover rate among nurses was also high [14,15].

The aim of this study was to describe how the lockdown affected elderly homecare patients. We present a combination of the results of the structured screening questions and the informant’s open-ended responses. The study participants were community-dwelling older adults, most with some cognitive or functional impairment and in urgent need of further assessment. Five topics illustrated the participants’ thoughts and moods during the lockdown: (a) satisfied; (b) lonely; (c) depression; (d) worried of COVID-19 infection; and (e) death and dying.

### 4.1. Challenges Facing Homecare in Lockdown

The homecare nursing staff were unprepared and stressed by the pandemic. This generation of nurses had never been confronted with a contagious virus such as COVID-19 and were unfamiliar with infection suits, masks, and gloves. With this equipment, it seemed difficult for the nurses and their patients to maintain a good relationship. The patients were worried. Nursing students who usually assisted nurses were not wanted anymore and restricted to online studies [26]. The staff were experiencing increased stress, anxiety, and fear of contracting the infection. For the first few weeks, the staff had no or very little knowledge about how to identify patients at risk. They had little information about an interim guidance for homecare for patients with suspected or confirmed COVID-19 and management of their contacts [27]. The guidelines advised health personnel in practical nursing on how to inform families who took care of relatives at risk. The relatives and friends were instructed to reduce or change their contact with other people to avoid transmitting the virus. Falatah [28] emphasized in her review the recommendation to improve the nurses’ competencies in caring for patients with COVID-19, which might reduce psychological factors and thereby reduce turnover rates. We believe that better competence will also enable nurses to identify patients at high risk for COVID-19, patients with possible COVID-19 and in need of testing, how to care patients with COVID-19, and the persons in their household. Better knowledge about the virus and the disease will enable nurse to help stop the spread of virus.

The staff attempted to organize the homecare personnel to visit the same patients at fixed times. However, the whole situation became unstable, and it was difficult to maintain schedules. Several of the patients needed daily assistance to get out of bed, perform personal hygiene, showering, dressing, food, medication, and other nursing activities. Some of the patients needed help several times, day and night, from one or two persons. The elderly and their relatives were afraid of letting other “strangers” into their home and felt uneasy when a new person from the homecare team arrived. They feared that they were bringing the COVID-19 virus into their homes. They were also tired of repeatedly teaching new personnel about their special routines. Some of the participants missed the opportunity for exercise outdoors, or weekly visits to a physiotherapist. The “lockdown” situation gave little room for more than primary care. Sama et al. [15] carried out a survey in Massachusetts and observed that both nursing and homecare services decreased during the pandemic. In Norway, the homecare service increased their use of digital aids, mainly for consultations and medications.

### 4.2. Effect of Lockdown on Homecare Clients

All of the participants had stories about how COVID-19 affected their lives. The combination of needing homecare and feeling isolated led to depression. Whitehead et al. [16] studied what factors caused elderly (60+) stresses and joys. Restrictions and confinement were the most common sources of stress. After the “lockdown” in spring 2020, the elderly became isolated. They could not meet family and friends who were their highest sources of joy [29]. The majority of elderly did not have a regular visitor service. Many were tired and had no strength to invite former friends, which worsened their mental health situation. Due to the risk of infection, the elderly did not dare to leave their homes.

“*I miss being able to take the tram to the city, drinking coffee with friends, walking up the main street, looking at books in the bookstore*”. Most of the people in this sample missed their normal daily schedule from before the lockdown. They missed visits and activities outside the home as it used to be.

Food supplies were also a serious concern for both close relatives and homecare services who assisted the elderly [30]. Some of the homecare patients suffered from diseases that caused difficulties in breathing, moving around, had a tendency to fall, and suffered from pain or other serious symptoms. This led to inactivity and impaired functioning. Older people are often not able to notice early signs of physical, cognitive, or mental health problems. Two thirds (*n* = 20) of the participants scored high on the assessment urgency algorithm due to the presence of cognitive or functional impairments. The participants had one or more symptoms that could be a sign of COVID-19. Since these current interviews was in the first year of the Norwegian lockdown, the symptoms could either be an ordinary influenza or stomach trouble. However, all small signs of any kind of illness disturbed the elderly and made them anxious. The high numbers of infected elderly were frightening. Several participants expressed worry that they would have to move into a nursing home. One daughter brought her mother back home after the onset of the pandemic.

The current situation created anxiety and the participants expressed uncertainty about the future. Isolation and long days were hard for the elderly to endure. The strict rules concerning isolation reduced their energy. Several of the participants gave an impression of having had a long and meaningful life. Due to the uncertain future, the thought of death was not frightening. Several expressed that they were not afraid to die but hoped they would be able to obtain sufficient palliative care and not be alone when death approached. However, the same persons hoped for a new vaccine and to be able to resume normal life.

### 4.3. What Contributes to Having a Good Life in Lockdown?

The patients that lived with a spouse emphasized the importance of being two persons in the household. Most of the participants lived alone. Family, friends, and helpers contributed to making the patient’s life meaningful. Several visits took place outside at the recommended distance, others met inside with their own little group. To carry out normal activities gave meaning to life such as having a meal together, assisting with grocery shopping, and getting medications at the pharmacy.

The patients living alone compensated for a lack of visits by calling friends and family.

A couple of patients had agreements with friends to check on them if they did not respond to calls. Several experienced joy to talk with nurses that had roots from different countries, eager to learn about their culture. Other activities such as listening to the radio and TV programs, Internet activities, reading books, and newspapers gave meaning. Other patients enjoyed listening to music and playing an instrument and activities such as knitting and thinking of fond memories from their childhood.

### 4.4. Implications

Both the World Health Organization and most governments recommended that the entire human population should ‘stay-at-home’ for some period to prevent the spread of COVID-19 during the pandemic. However, to reduce loneliness, local communities needed to organize a comprehensive approach. Different groups including charities, organizations, and healthcare providers should work together “*to support older people through this period of social isolation and to minimize and mitigate the negative impact of negative ageism, social isolation, and loneliness*”, as emphasized by Brooke and Jackson [31]. Elderly people form a mixed group. Some of them love music or reading, while others enjoy watching television. However, overall, their physical activities seemed to be rather low. The authorities recommended that during periods of lockdown, physical exercise should be as vigorously promoted as social distancing itself [32]. Twelve (40%) of the patients needed help for activities of daily living. However, without a partner or a friend, it seems that many elderly became more passive, waiting for the homecare staff.

This study contributes to drawing a picture of elderly people living at home in Norway who receive home nursing care. We initiated this study very shortly after the pandemic occurred. As researchers, we saw it as an important contribution to society to map the experiences of some of our society’s most vulnerable inhabitants. Qualitative methods can yield findings that are closer to the respondents than larger quantitative studies. It is therefore a separate strength in itself to conduct personal interviews with the vulnerable. In retrospect, we can see that our main findings are in line with other and recent research in this field [33,34]. This is how our research fits within a larger narrative about how the pandemic affected the elderly population.

In the future, a screener such as the interRAI CVS self-report survey used in this study could be used to assess for needs in older homecare patients and could provide the nursing teams with information in the initial stages of infection. This would heighten the quality of care and prevent the spread of infection.

## 5. Conclusions

In March 2020, a worldwide pandemic threatened people all over the globe. The virus spread from person to person, town to town. Older people were at greater risk. Isolation seemed to be the best weapon in reducing the spread of infection. This current study used a structured screening assessment. The questions activated positive and negative feelings from the homebound elderly. Homecare nurses struggled to meet the needs of the homebound elderly in lockdown. Nevertheless, during this time, the participants were able to adjust their situation and obtain food and medical care. However, missing their close contact with family and friends was challenging. The fear of becoming infected and perhaps dying made several of them uneasy, while others were ready to die. Community-based providers and organizations should work together to identify unmet physical and social needs among the elderly living in the community and organize a comprehensive approach to respond appropriately.

## Figures and Tables

**Table 1:** Description of elderly homecare client characteristics as reported using the interRAI COVID-19 Vulnerability Screener (*N* = 30).

Characteristics	*N* = 30 (%)
Female	19 (63.3)
Male	11 (36.7)
Age (Md + SD) Female	83.0 (11.5)
Age (Md + SD) Male	81.1 (8.1)
Lives alone	21 (70.0)
Coronary heart disease or high blood pressure	15 (50.0)
Chronic obstructive pulmonary disease (COPD; included two with asthma)	7 (23.3)
Reports one or more possible COVID-19 symptoms	15 (50.0)
Elevated or high risk of mortality due to COVID-19 (MCC score ≥1; scale 0–2)	22 (73.3)
Lonely occasionally or more often (score ≥2; scale 0–4)	15 (50.0)
Feels depressed or hopeless often, or daily (score ≥1; scale 0–3)	17 (56.7)
Count of impairments in ADLs (ADL-CT score ≥1; scale 0–4))	12 (40.0)
Cognitive and or functional impairment (AUA score ≥5; scale 0–6)	20 (66.7)
Answered all the questions by myself (the informant)	24 (80.0)

**Table 2:** Central categories, descriptions, and quotes.

Category	Descriptions	Quotes
Satisfied (Seven participants)		
Close family	When the family supports well, the elderly feel more secure. Living with a spouse makes isolation easier.	F, 84 years. “*I do not feel lonely. It is good to live with my spouse, the children assist us.*”
Contact via telephone/Internet	Mobile telephones and the Internet functioned well for several of the participants. However, the Internet was not a realistic option for the oldest old	M, 77 years. “*I have a daughter in USA, and we keep contact via Internet.*” “*I order food via the internet and the nurses take care of it.*”
Appreciates homecare	Most of the participants expressed that they were satisfied with the homecare service. However, they would like the staff to be more consistent.	F, 73 years. “*I am very happy with the homecare service even though it is stressful with all the different people.*”
Lonely (Nine participants)		
Few visitors	The next-of-kin were afraid they would infect the respondent. The homecare service was often the only visitors.	F, 87 years. “*It has been sad not to see grandchildren and great-grandchildren for several months.*”
Depression (Seventeen participants)		
	Some patients are unable to dress themselves and get out for a short walk. The combination of needing homecare and feeling isolated led to depression.	F, 78 years. “*I had intended to reduce the antidepressants, but then came the coronavirus, and I became even more depressed.*”
Worried about COVID-19 (Eleven participants)		
Little knowledge about COVID-19	Neither the leaders nor the homecare staff had sufficient knowledge and equipment to meet the demands of the pandemic.	F, 87 years. “*I am feeling insecure. I have been in a two-week quarantine. One of my nurses had a positive COVID-19 test.*”
Terrified of infection	Some elderly needed homecare several times a day. The providers’ knowledge and use of protection equipment could be somewhat unstable as well as infection regime.	F, 73 years. “*I am terrified of being infected of the COVID-virus and transferred to a hospital.*”
Death and dying (Six participants)		
When time is up	Some of the participants expressed that death and dying did not seem frightening. When their time is up, they want someone to be there and give sufficient palliative care.	F, 91 years. “*I do not understand why I should live any longer. I feel like I’m ready to die.*”

*Note*. F = Female; M = Male.

## Data Availability

The data presented in this study are available on request from the corresponding author. The data are not publicly available because informed consent was given for this specific study and the data cannot be used for secondary purposes.
